# Combining Virtual Screening Protocol and In Vitro Evaluation towards the Discovery of BACE1 Inhibitors

**DOI:** 10.3390/biom10040535

**Published:** 2020-04-01

**Authors:** Judite R. M. Coimbra, Salete J. Baptista, Teresa C. P. Dinis, Maria M. C. Silva, Paula I. Moreira, Armanda E. Santos, Jorge A. R. Salvador

**Affiliations:** 1 Laboratory of Pharmaceutical Chemistry, Faculty of Pharmacy, University of Coimbra, 3000-548 Coimbra, Portugal; judite.coimbra@student.ff.uc.pt (J.R.M.C.); msilva@ff.uc.pt (M.M.C.S.); 2 Center for Innovative Biomedicine and Biotechnology (CIBB), Center for Neuroscience and Cell Biology (CNC), University of Coimbra, 3004-504 Coimbra, Portugal; saletejbaptista@gmail.com (S.J.B.); tcpdinis@ci.uc.pt (T.C.P.D.); pismoreira@gmail.com (P.I.M.); aesantos@ci.uc.pt (A.E.S.); 3 Chem4Pharma, Edifício IPN Incubadora, 3030-199 Coimbra, Portugal; 4 Laboratory of Biochemistry and Biology, Faculty of Pharmacy, University of Coimbra, 3000-548 Coimbra, Portugal; 5 Laboratory of Physiology, Faculty of Medicine, University of Coimbra, 3000-548 Coimbra, Portugal

**Keywords:** Alzheimer’s disease (AD), amyloid-β (Aβ), BACE1, small bioactive molecules, virtual screening, pharmacophore modelling, molecular docking, structure- and ligand-based methods

## Abstract

The treatment options for a patient diagnosed with Alzheimer’s disease (AD) are currently limited. The cerebral accumulation of amyloid-β (Aβ) is a critical molecular event in the pathogenesis of AD. When the amyloidogenic β-secretase (BACE1) is inhibited, the production of Aβ peptide is reduced. Henceforth, the main goal of this study is the discovery of new small bioactive molecules that potentially reach the brain and inhibit BACE1. The work was conducted by a customized molecular modelling protocol, including pharmacophore-based and molecular docking-based virtual screening (VS). Structure-based (SB) and ligand-based (LB) pharmacophore models were designed to accurately screen several drug-like compound databases. The retrieved hits were subjected to molecular docking and in silico filtered to predict their ability to cross the blood–brain barrier (BBB). Additionally, 34 high-scoring compounds structurally distinct from known BACE1 inhibitors were selected for in vitro screening assay, which resulted in 13 novel hit-compounds for this relevant therapeutic target. This study disclosed new BACE1 inhibitors, proving the utility of combining computational and in vitro approaches for effectively predicting anti-BACE1 agents in the early drug discovery process.

## 1. Introduction

Alzheimer’s disease (AD) is a neurodegenerative disorder that massively alters the mental capacity of patients suffering from this condition. The neurodegenerative process is associated with extensive neurotransmission deficits and neuronal function impairment in cerebral areas essential to cognitive processes, being responsible for the clinical manifestation of dementia [1]. Despite the advanced research in AD, in terms of understanding the pathological process and identifying biomarkers for diagnosis and key targets for therapy [2], there is still no effective pharmacological treatment to prevent the advance of the disorder [3]. Hence, it is important to pursue the development of disease-modifying therapies acting the main targets involved in the disease’s pathogenesis allowing to slow the development of structural and functional abnormalities in the central nervous system (CNS), which could potentially provide sustainable improvements of cognitive functions [4].

The cerebral accumulation of the misfolded amyloid-β (Aβ) oligomers have been indicated as the main incident underlying neurotoxic events in AD [5]. The formation of Aβ peptide requires the initial cleavage of Amyloid Precursor Protein (APP) by the transmembrane aspartyl protease BACE1 (β-site APP cleaving enzyme 1), which is one of the major therapeutic targets being currently explored for an AD-modifying intervention. Although BACE1 inhibition is recognized worldwide as a potential strategy in decreasing Aβ neurotoxic concentrations [6], the development of effective BACE1 inhibitors has been a demanding process [7]. In order to reach the brain, the inhibitors need to cross the blood–brain barrier (BBB) and neuronal membranes, thus a clear strategy to target BACE1 is based on small molecules, which are more prone to exhibiting a broad range of suitable drug-like properties [8].

The BACE1 enzymatic domain is folded in two symmetrical N- and C-lobes that are divided by a cleft containing in the center the two catalytic aspartate residues—Asp32 and Asp228—that mediate the substrate proteolytic cleavage mechanism of this protease [9]. The active site is partially capped by a flexible β-hairpin loop known as the flap (residues 67–77) and it is formed by subpockets with different properties in terms of polarity, for instance, it is noted large a hydrophobic cleft formed by the side chains of Leu30, Ile110, Ile118 (aliphatic residues), Tyr71, Phe108, and Trp115 (aromatic residues) and subsites more solvent-exposed and hydrophilic [9,10]. Moreover, due to the large size of catalytic domain that hinders the design of potent small inhibitors, some important structural features should be taken into consideration to enhance the binding affinity within key residues of the binding pocket, particularly the catalytic aspartic dyad [9,10]. Interactions with Tyr71, Thr72, and Gln73 (flap region), among other residues (Gly11, Gly34, Gly230, Thr231, Thr232, and Arg235) have also been reported as crucial for the inhibition activity of a wide variety of scaffolds of small BACE1 inhibitors that have been developed in the last years [11]. Experimental data have also shown that the cellular trafficking of BACE1 involves different subcellular acidic compartments at the endosomal system, suggesting that its subcellular distribution is also a key determinant of its activity [12]. Therefore, an additional strategy to address the inhibitors is to ensure they will be optimally protonated to efficiently inhibit the enzyme at a mildly acidic pH, which favors BACE1 activity [13].

Although BACE1 inhibition could effectively halt the production of Aβ and its accumulation on plaques (one of the hallmarks of AD), over the past years, none of the BACE1 inhibitors tested in clinical trials has been approved so far [14]. Current explanations confirmed that many anti-BACE1 drugs’ failure is not based neither on the inefficacy nor other pharmacokinetic limitations but mainly on safety concerns, which has been indicated as the major barrier against the success across different clinical trials [15]. Furthermore, according to recent preclinical evidence, the timing of BACE1 inhibitors administration in clinical testing should be adapted for early stages of AD when BACE1 inhibition is expected to have the highest impact in preventing amyloid plaque deposition [16,17]. Thus, these results suggest that BACE1 inhibitors may not be expected to induce cognitive improvements in patients already exhibiting severe AD symptoms but are very effective in patients at preclinical AD (seemingly healthy with no main symptoms), including those in which biomarkers levels related to AD are high [2]. However, the chronic clinical use of BACE1 inhibitors needs caution since the enzyme has relevant physiological functions required for optimal synaptic function such as axon growth, myelination, neurogenesis, and synaptic plasticity [18]. Besides, BACE1 processes other cellular substrates beyond APP, such as Neuregulin1 (NRG1), close homologue of L1 (CHL1), and seizure-related gene 6 (SEZ6) [18]. Fortunately, studies suggest that partial inhibition of BACE1 activity might be enough to prevent amyloid pathology and reduce the risk of developing AD [19].

**Figure 1:**
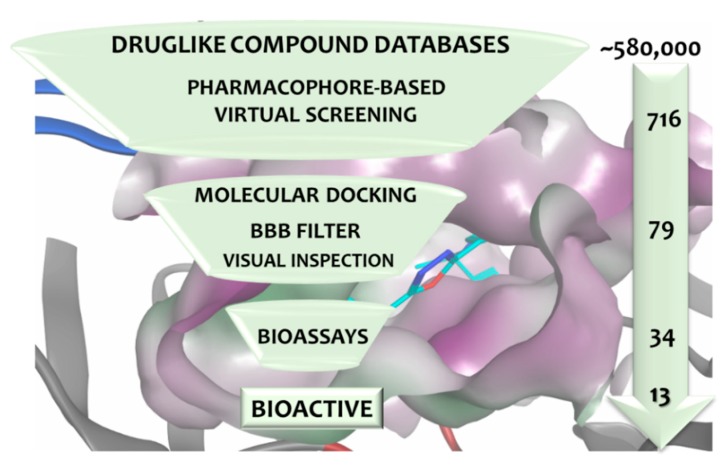
The virtual screening (VS) workflow applied to identify novel BACE1 (β-site APP cleaving enzyme 1) hit inhibitors. The number of compounds remaining after filtering at each stage is listed.

The present work illustrates the application of molecular modelling tools to identify novel BACE1 inhibitors. Molecular modelling consists of well-established computational methodologies that speed up, rationalize, and balance the costs of the drug discovery process [20]. The combination of these powerful methods in a virtual screening (VS) strategy allows to dramatically reduce the number of candidates for experimental validation [21]. The multiple-step VS protocol included the construction and rigorous validation of structure-based (SB) and ligand-based (LB) pharmacophore models used afterwards to search drug-like databases to potentially identify small molecules acting on BACE1. Subsequently, molecular docking studies were carried out to predict binding modes and affinities of the retrieved compounds. Additionally, an in silico filter to predict brain access, which is crucial for these drug candidates, was also applied. After a visual examination of the binding poses, 34 compounds were further selected for biochemical evaluation. Finally, a set of new structural compounds was found to moderately inhibit BACE1. The workflow of this study is illustrated in Figure 1.

## 2. Materials and Methods

### 2.1. Dataset Preparation

The compound datasets—the National Cancer Institute (NCI), Drugbank, Asinex, Specs, and Chembridge—were downloaded and filtered by the *Lipinski Druglike Test* descriptor available in MOE 2016.0802 software package (Molecular Operating Environment, Chemical Computing Group, Montreal, Canada) [22]. This filter is based on Lipinski’s rule of five, which ensures oral bioavailability of compounds [23]. The final curated database comprises ~580,000 unique chemicals. Training and Test set for the pharmacophore modelling comprised known BACE1 inhibitors, whose chemical structures and experimental data were collected from ChEMBL Database Release 23 (ChEMBL23, 10.6019/CHEMBL.database.23 accessed 22 May 2017) [24]. Therefore, only compounds with IC_50_ <1000 nM for BACE1 inhibition Fluorescence Resonance Energy Transfer (FRET) assay (single protein format) resulting from the search for known BACE1 inhibitors on ChEMBL database were further considered on the construction of these sets. For pharmacophore validation, 276 BACE1 active compounds and 17741 decoys (inactive compounds) obtained from the Database Useful Decoys Enhanced (DUD_E) (http://dude.docking.org/ accessed 19 May 2017) [25] were also used.

To prepare the datasets mentioned above, the compound structures were first converted into Molecular database format (.mdb) and the conformations of the ligands were generated by the Conformation Import application in MOE with an imposed limit of 4.5 kcal/mol strain energy and a maximum of 500 conformations per molecule. The wash setting was applied at pH 6.0, hydrogen atoms were added, and protonation states were assigned. The stereochemistry and tautomerization remained the same as from the original database. Finally, the minimum energy configuration was calculated using the MMFF94x force field.

### 2.2. Structure-Based (SB) Pharmacophore Modelling

The SB-pharmacophore models were obtained from the structural data of four BACE1-ligand complexes retrieved from Protein Data Bank (PDB ID: 2WF1, 2QMF, 2IRZ, and 4ACU) using the Protein Ligand Interaction Fingerprints (PLIF) tool implemented in MOE 2016.0802 software package [22]. All the crystal structures of BACE1 were prepared using Structure Preparation application, protonated (at pH 6.0 and 300 K) using the Protonate 3D tool, and hydrogen atoms were added. OPLS-AA force field [26] was used to assign atom types and partial charges to each atom in the receptor structure, which was further energy-minimized using the same force field. These complexes were chosen based on their structural reliability, the chemical variety of the potent co-crystallized ligands, which represent some of the first- and second-generation classes of BACE1 inhibitors and due to the several types of interactions observed within the active site (useful for the PLIF application purpose). The PDB complexes were aligned and superposed using the Sequence Editor. The PLIF application converts the homogeneous set of interaction fingerprints into pharmacophoric features. The feature coverage was set at more than 50% and a maximum radius of 3.0. All other options remained at the program’s default.

### 2.3. Ligand-Based (LB) Pharmacophore Modelling

**Figure 2:**
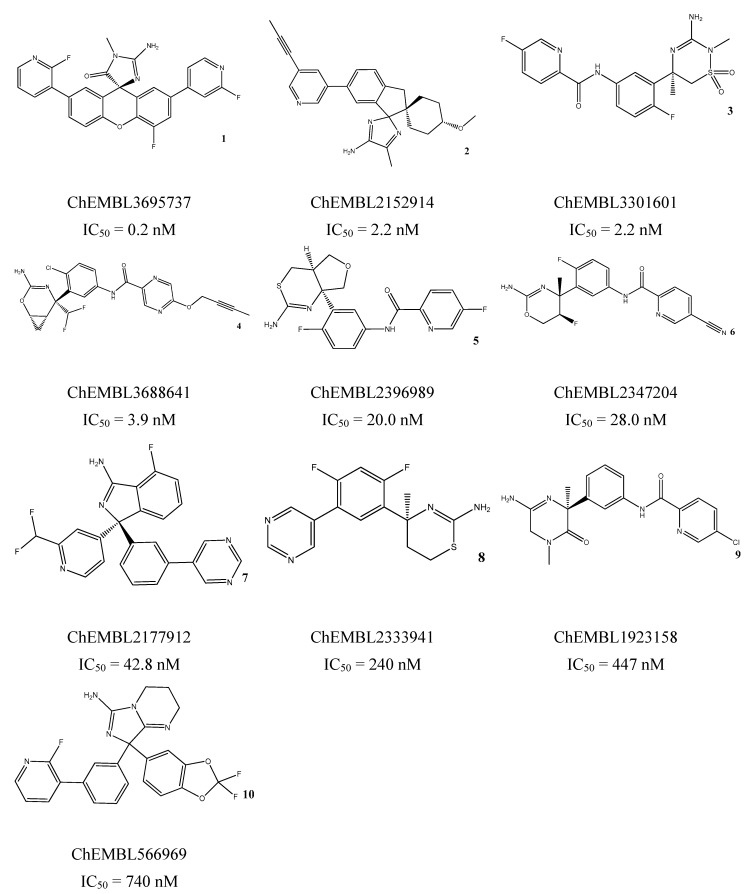
Chemical structures of the Training set used for mapping of consensus pharmacophoric features for the ligand-based (LB) approach with an activity range (IC_50_ value) from 0.2 nM to 740 nM.

The LB-pharmacophore models were determined from ten potent known BACE1 inhibitors representing different classes of inhibitors (ChEMBL ID: 3695737, 2152914, 3301601, 3688641, 2396989, 2347204, 2177912, 2333941, 1923158, and 566969) (compounds **1**–**10** in Figure 2) using the Pharmacophore Elucidation tool implemented in MOE 2016.0802 [22]. The application exhaustively searches for pharmacophore models that induce good overlay of most of the Training molecules. Therefore, two types of pharmacophores were generated based on the parameter’s selection—the Query Spacing was set to 0.8 for Elucitade_1 and to 0.6 for Elucitade_2. In the first phase of pharmacophore query generation, all possible queries are considered such that the inter-query distances are placed on a gird with multiples values of the supplied Query Spacing value (larger values lead to coarser queries and too small values may lead to a few common queries) and since it can generate a great many queries that have very similar geometries, they are clustered to reduce their number. The Query Cluster value specifies the root-mean-squared deviation (RMSD) value (in angstroms) used to cluster the queries before overlap and classification scoring. For both, the Query Cluster was set to 0.75, the MMFF94x was the Forcefield applied, the Feature limited value (the maximum number of features per pharmacophore) was 6, and the scheme was modified in the following way: the feature hydrogen bond donor (*HBD*), hydrogen bond acceptor (*HBA*), and aromatic ring (*Aro*) with 1.8 of radii were added; the radii of projection of *HBD* (*PHBD*) and *HBA* (*PHBA*) were set to 1.0. All other parameters remained at the program’s default. The output overlapping value (the score of the alignment) varies between [0, *n*] where *n* is 10, the maximum number of active molecules, and the cover value is the number of actives molecules that match the query. Higher values indicate better alignments and coverage.

### 2.4. Validation of Pharmacophore Modelling

In the VS field, the quality of a model can be assessed by a few metrics. The following validation sets were employed for the pharmacophore modelling validation—the Training set, the Test set, and the actives and decoys from DUD_E. The Training Set for the SB model contained the four co-crystallized ligands (ChEMBL ID: 539436, 403727, 219601, and 3260842) of the PDB complexes: 2WF1, 2QMF, 2IRZ, and 4ACU (Supplementary Figure S1); the Training Set for the LB model comprised the ten ligands above mention (Figure 2). The Test Set is composed by 39 structurally diverse small molecules (Supplementary Figure S2) with an IC_50_ value from 0.3 nM to 1000.0 nM against BACE1 (at the same biological assay); two different categories were created: high actives (0.3–699 nM) and moderate actives (above 700 nM). The ligands of each Training Set were included in the Test Set of the other approach. Thus, the Test Set of the SB approach has a total of 49 ligands, and the Test Set of the LB approach contained 43 ligands. The hit percentage of each set was measured, and the retrieved compounds were classified according to their RMSD value, which means the root of the mean square distance between the query features and the matching annotation points of the molecules. Lower RMSD values indicate better quality of the matching of the molecule to the pharmacophore model. Further analysis allowed to correlate this raking with the reported experimental activity.

The predictive nature of the pharmacophore models was assessed by using the actives and decoys. The accuracy (Equation (1)) of the in silico classifications (the proportion of compounds correctly identified as active or inactive in the dataset) was calculated by

(1) $$\%\;\mathrm{Accuracy}\;=\frac{\mathrm{TP}\;+\;\mathrm{TN}}{D}$$

where True positive (TP) is the number of BACE1 inhibitors correctly identified as actives, True negative (TN) is the number of non-BACE1 inhibitors correctly identified as inactives and D is the total number of compounds in the validation dataset. The sensitivity (Se) (Equation (2)), which expresses the ability of the model to correctly identify active compounds from all the actives and the specificity (Sp) (Equation (3)), which reflects the ability of the model to correctly identify inactive compounds from all inactives in the dataset, were calculated by

(2) $$\%\;\mathrm{Sensitivity}\;\left(\mathrm{Se}\right)=\frac{\mathrm{TP}}{\mathrm{TP}\;+\;\mathrm{FN}}$$

(3) $$\%\;\mathrm{Specificity}\;\left(\mathrm{Sp}\right)=\frac{\mathrm{TN}}{\mathrm{TN}\;+\;\mathrm{FP}}$$

where False negative (FN) is the number of BACE1 inhibitors incorrectly identified as inactive and False positive (FP) is the number of non-BACE1 inhibitors incorrectly identified as actives. The retrieved molecules of the validation set were ranked based upon their fitness score (RMSD). These lists were then used to calculate the enrichment factor (EF), described in Equation (4). The overall enrichment expresses the probability that an active compound is ranked higher than a decoy. As the early portion of the hitlist is of greater interest as well, a metric more sensitive to measure the early enrichment at 0.5% of the ranked database screened—the EF_0.5%_—was also employed (Equation (5)) [27,28]. The method shows a positive enrichment to better detect active compounds than random selection when the EF value is more than 1.0 [28].

(4) $$\mathrm{Enrichment}\;\mathrm{factor}\;\left(\mathrm{EF}\right)=\frac{\mathrm{TP}\;\times \;D}{\mathrm{Ht}\times A}$$

(5) $$\mathrm{Enrichment}\;\mathrm{factor}\;\mathrm{at}\;0.5\%\;\left(\mathrm{EF}\;0.5\%\right)=\frac{\mathrm{TP}\;0.5\%\;\times \;D}{\mathrm{Ht}\;0.5\%\;\times \;A}$$

where A is the total number of active compounds in the dataset, TP_0.5%_ is the number of active compounds in the 0.5% of the ranked dataset, Ht is the total number of compounds retrieved by the model, and Ht_0.5%_ is the number of compounds in the 0.5% of the dataset.

Finally, these lists were then used to calculate the percentage of known actives (TP) found vs. a fixed percentage of false positives (0.5%, 1%, 5%, and/or the total of FP), and the Receiving Operating Characteristic (ROC) plots (made in Microsoft Excel), which represent the fraction of TP recovered (Se) vs. the fraction of FP recovered (1-Sp).

### 2.5. Pharmacophore-Based Virtual Screening and Molecular Docking

The SB and LB pharmacophore models were used to quickly screen the compound databases using the Pharmacophore Search setting of MOE with total match mode selected. The molecular docking simulations were performed using GOLD suite v5.2.2 software (The Cambridge Crystallographic Data Centre (CCDC), Cambridge, UK) [29,30]. For the docking studies, the crystal structure of human BACE1 was retrieved from Protein Data Bank (PDB ID: 2QP8) and prepared using MOE [22]. The chain B and water molecules were removed from the system and the receptor structure was protonated (at pH 6.0 and 300 K) using the Protonate 3D tool and hydrogen atoms were also added. OPLS-AA force field [26] was used to assign atom types and partial charges in the receptor structure, which was further energy-minimized using the same force field. The active residues were defined by considering the binding site residues, previously identified by the Site Finder tool on MOE package. The subsequent steps were performed in GOLD. The crystal ligand was removed, the GA Runs were set to 500 (for docking-based VS) and 1000 (for redocking) and the option “allow early termination” was deselected. ChemPLP scoring function was chosen in slow most accurate mode, with 100% of search efficiency to proceed with the simulations. For each molecule, the ten best solution poses were kept. The remaining default parameter settings were used for docking calculations.

The docking protocol was firstly validated through self-docking. The ligand of the 2QP8 complex was docked back into the corresponding protein structure using all docking-scoring functions available in GOLD. As a measure of docking reliability, the RMSD value was used to compare the differences between the atomic distances of the docked poses and the co-crystallized pose, where a threshold of 2.0 Å is widely accepted as corresponding to good docking solutions [31]. The predicted orientations and binding modes were then visualized using MOE.

### 2.6. Blood–Brain Barrier Penetration Prediction

The retrieved hits by the pharmacophores were filtered by a tool in the web service SwissADME [32] (http://www.swissadme.ch/, Swiss Institute of Bioinformatics, Lausanne, Switzerland), to predict which compounds are expected to cross the BBB. The former software uses physicochemical descriptors which allow predicting pharmacokinetic properties, among others, to support drug discovery. To compute the brain penetration prediction, a methodology described as BOILED-Egg (Brain Or IntestinaL EstimateD permeation) was applied by the server [33]. This model is based on the calculation of lipophilicity (logP) and polarity (topological polar surface area (tPSA). Molecules with moderate polarity (PSA <79 Å) and lipophilicity (logP from +0.4 to +6.0) have a high probability to cross the BBB by passive diffusion and access the CNS [33].

### 2.7. Cell-free Assay for BACE1 Activity

The compounds tested in this work were purchased from Specs (Zoetermeer, The Netherlands), Asinex (Delft, The Netherlands), and Chembridge (San Diego, CA, USA) or supplied by NCI (Rockville, MD, USA). BACE1 inhibition was evaluated by using an in vitro cell-free screening assay using the β-Secretase (BACE1) Activity Detection Kit CS0010 (Sigma-Aldrich Co. LLC., St. Louis, MO, USA). In short, the BACE1 enzyme solution was pre-incubated with different concentrations of the tested compounds for 15 min at 37 °C followed by the addition of the BACE1 substrate. Then, according to the manufacturer’s protocol, the reaction was performed for 2 h at 37 °C. The assay is based on the FRET method and the signal was measured with a spectrofluorometer (Fluorimeter SpectraMax Gemini EM, Molecular Devices, LLC., San Jose, CA, USA) at excitation and emission wavelengths of 320 nm and 405 nm, respectively. The effect of the inhibitor concentration on enzyme activity was evaluated by the percentage of enzyme inhibition relative to the maximum enzyme activity determined in the absence of inhibitors. A commercial inhibitor of reference (β-Secretase Inhibitor IV, Calbiochem, Merck KGaA, Darmstadt, Germany) was also used as a positive control on each independent assay [34].

### 2.8. Descriptors

The physicochemical properties of the active hits, including molecular weight (MW), the logarithm of the octanol/water partition coefficient (logP_o/w_), and polar surface area (PSA) were computed using MOE molecular descriptors.

## 3. Results

### 3.1. Structure-Based Pharmacophore’ Generation

**Figure 3:**
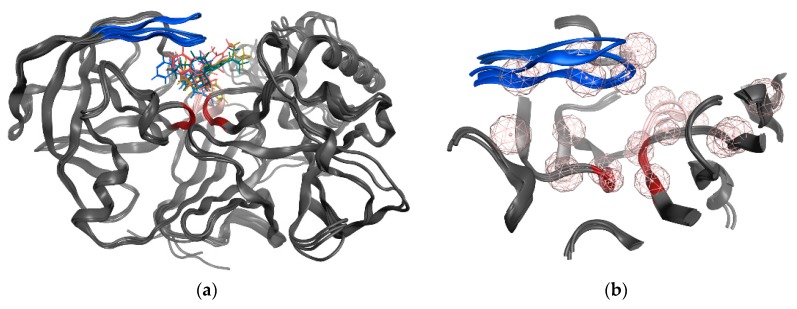
(**a**) Superposition of the four BACE1 complexes for generating common pharmacophoric features based on the interactions observed in the crystal structures. (**b**) Twenty-three excluded volume (*ExcV*) (light rosy spheres) are present in all structure-based (SB) models. The colors red, blue and light pink represent the catalytic motif, the flap region, and the 10s loop of the BACE1 protein, respectively.

The SB pharmacophore modelling was determined from the structural data of four BACE1-ligand complexes retrieved from PDB (ID: 2WF1, 2QMF, 2IRZ, and 4ACU). The four BACE1 complexes were aligned and superimposed (as shown in Figure 3a) and the following homogeneous set of ligand-protein interaction fingerprints were identified: Asp32 and Asp228 sidechain hydrogen bond acceptor (*HBA*); ionic interactions with Asp228; Thr232 backbone hydrogen bond donor (*HBD*); Gly230 backbone *HBA*; hydrophobic (*Hyd*) interactions with Gly230; Gln73 backbone *HBD*; Gly34 backbone *HBA*; and π-interactions type with Thr231 (Supplementary Figure S3 shows all ligand-receptor interactions identified in the four PDB complexes). Afterwards, six consensus pharmacophoric features derived from the set of protein-ligand interaction fingerprints were retrieved by the PLIF application—two hydrogen bond acceptors and metal ligation (*HBA&ML*), one located in the hydrophilic pocket formed by Thr232, Asn233, Arg235, and Ser325 and the other near to Thr72 and Gln73 at the flap region; one *Hyd* feature located in the hydrophobic cleft formed by the side chains of Leu30, Phe108, Trp115, and Ile118; one *HBD* in the vicinity of Gly230; one mixed-type feature (*HBD&HBA&ML*) near Asp32; and one hydrogen bond donor and cation (*HBD&Cat*) representing interactions with Asp32, Gly34, and Asp228. These pharmacophoric features were edited using the pharmacophore Query Editor, to optimize their radii and/or add new features. The refined pharmacophores contained the replacement of one of the *HBA&ML* features by *HBA*, representing the interactions within the subpocket formed by Thr232, Asn233, Arg235, and Ser325; an additional projected point of hydrogen bond donor (*PHBD*), which represents the interaction with the catalytic Asp228; and an aromatic ring center (*Aro*) representing the aromatic interactions between Thr231 and/or Thr232. Additionally, twenty-three excluded volumes (*ExcV*), translating the protein regions of the binding site (concordant in all complexes), which do not allow any matching atoms inside during the screening process, were included in the model (Figure 3b). Finally, several pharmacophore hypotheses with different combinations of the features mentioned above were generated and the best three combinations (SB_Hyp1, SB_Hyp2, and SB_Hyp3) are shown in Table 1.

### 3.2. Ligand-Based Pharmacophore’ Generation

The generation of pharmacophore models based on ligands was obtained from the common physicochemical properties of ten structurally diverse and potent BACE1 inhibitors (Figure 2). This approach allows determining consensus pharmacophoric features of the aligned Training molecules carried out in such a way that all of them satisfy the generated models. Hence, a collection of pharmacophore hypotheses was automatically retained by the application and the models with higher overlapping scores (better alignments) were chosen to refine. Using the pharmacophore Query Editor option, the original features were edited (radii optimized or deleted) and/or new features were added. The conserved features among all alignment hypotheses include the *HBD* associated with one or two *PHBD* and an *Aro* center. The molecular alignment, the original features and the respective refined model of the three best pharmacophores (LB_Hyp1, LB_Hyp2, and LB_Hyp3) are described in Table 2.

### 3.3. Validation of the Pharmacophore Modelling Protocol

**Figure 4:**
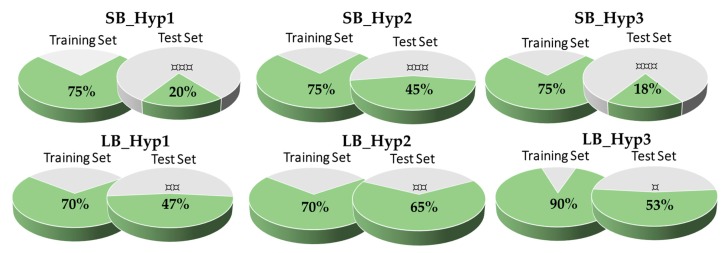
Pharmacophore model validation based on the hit’s detection on Training Set and Test Set. The pie charts show the percentage of retrieved BACE1 inhibitors. Dark grey color denotes relative correct classifications. The models that fit high actives with a lower root-mean-square deviation (RMSD) score (best fitness) and moderate actives with higher RMSD (worse fitness) are signaled by “¤¤¤”; the models that fit more high actives than moderate actives, are signaled by “¤¤”; the models that fit moderate actives with lower RMSD than the high active’s RMSD are signaled by “¤”.

The developed pharmacophore models were used to screen the compounds in the Training and Test datasets. The hit percentage of the Training and Test set for each model was calculated as showed in Figure 4. The retrieved Test set hits were sorted according to their pharmacophore-based fitness score (RMSD) and to evaluate if they represent the high or the moderate active compounds of the set. For the SB approach, the three models detect 75% of the Training set compounds and the hypothesis SB_Hyp1 and SB_Hyp3 have similar performance on the Test set, being SB_Hyp2 the model that identifies more hits in the Test set. For the LB approach, the hypothesis which has higher hit detection on the Training set and Test set is LB_Hyp3 and LB_Hyp2, respectively. In terms of the correlation between the experimental activity and the ranking position, SB models show a better correlation when compared to the LB models. The lowest correlation was shown for LB_Hyp3.

#### Enrichment Metrics

**Figure 5:**
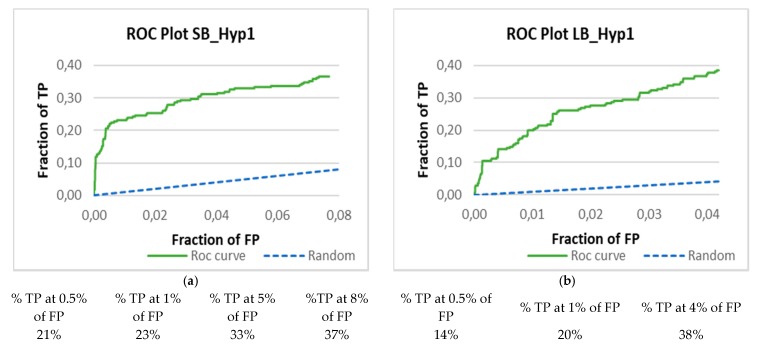
Receiver operating characteristic (ROC) curves and percentage of TP versus FP by the SB_Hyp1 (**a**) and LB_Hyp1 (**b**) (8% of the total database for SB_Hyp1 and 4% for LB_Hyp1). The curve only shows actives and decoys whose conformations match pharmacophoric requirements. No value is returned for molecules that do not match the pharmacophore constraints; therefore, they cannot be plotted. The green curve represents retrieval of active hits and blue is the expected performance by random guess.

A validation set containing actives and decoys was used to validate the developed hypotheses through a set of statistical measures, namely accuracy, sensitivity, specificity, and enrichment (Table 3). Analyzing the data from the SB approach, all models are highly selective and accurate (more than 90%). Among all pharmacophore models, SB_Hyp3 shows the lowest sensitivity (less than 30%). Moreover, it was observed more than 30-fold enrichment for finding actives in the top-ranked compounds (EF_0.5%_ value), except for the SB_Hyp2. For the LB strategy, all models show similar sensitivity (more than 30%), and all are highly selective and accurate (more than 90%). However, LB_Hyp1 was slightly better in these parameters, as well as in the enrichment of the top-ranked compounds that was approximately 23-fold. In Figure 5, it is shown the ROC plot and the true positive versus false positive profile of SB_Hyp1 and LB_Hyp1. When inactive fractions in the early regions of the ROC curve are selected, it can be noted a greatest early recovery performance in both cases; for SB_Hyp1, 21% of true positives at 0.5% of false positives (best profile of the SB approach), and for LB_Hyp1 14% of true positives at 0.5% of false positives (best profile of the LB approach) (Supplementary Table S1).

### 3.4. Virtual Screening

**Figure 6:**
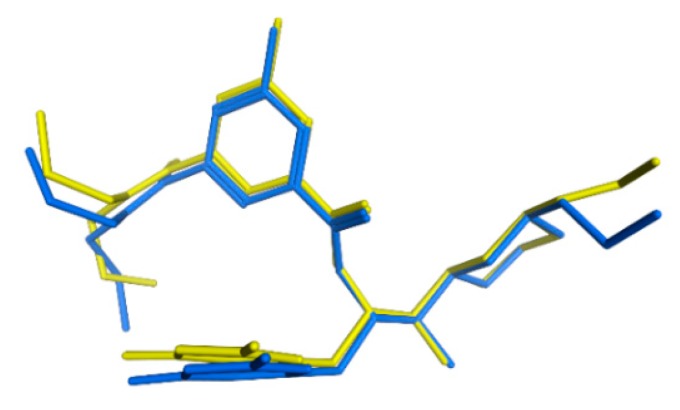
Self-docking validation. Superposition of the best-docked pose of ChEMBL257278 (0.544 Å) (blue) with its crystal structure conformation (yellow).

Based on the aforementioned validation methods, the SB_Hyp1 and LB_Hyp1 pharmacophore models were chosen to screen commercial datasets previously prepared. The pharmacophore-based VS identified 716 drug-like hits that match simultaneously the features of the two pharmacophore models. These hits were docked at the BACE1 binding site (PDB ID: 2QP8) using GOLD and were further subjected to a filter for estimating the BBB penetration using the web tool SwissADME [32]. According to the data from this online server, 79 compounds were predicted to cross the BBB. The docking procedure was validated through self-docking, as well as by docking 14 reference BACE1 inhibitors from the Training and Test datasets from the pharmacophore approach, considering all the available docking-scoring functions in GOLD. The best self-docking results were obtained for the ChemPLP scoring function, which is able to reproduce the binding pose of the co-crystallized ligand (ChEMBL257278) in the BACE1 crystal structure with an RMSD of 0.544 Å (Figure 6).

After the docking simulations, the compounds were ranked according to their corresponding docking scores. A cut-off of 55 was applied based on the score range of the 14 BACE1 inhibitors that were docked in the validation process ([121.00–55.74]), and the remaining compounds were redocked. To select promising compounds for in vitro assays, the binding poses and the predicted interactions within the active site of the top-scored compounds were visually inspected. The interaction with key residues of the active site, such as the aspartate catalytic dyad (Asp32 and Asp228) are privileged in precluding catalytic activity (since both residues mediate the catalytic reaction), among other interactions important in stabilizing the inhibitor within binding pocket, as well as the structural diversity between the molecules, were considered when choosing potential hits. The compounds were also analyzed for their commercial availability and their synthetic route access.

### 3.5. In Vitro Assessment of BACE1 Inhibition

**Figure 7:**
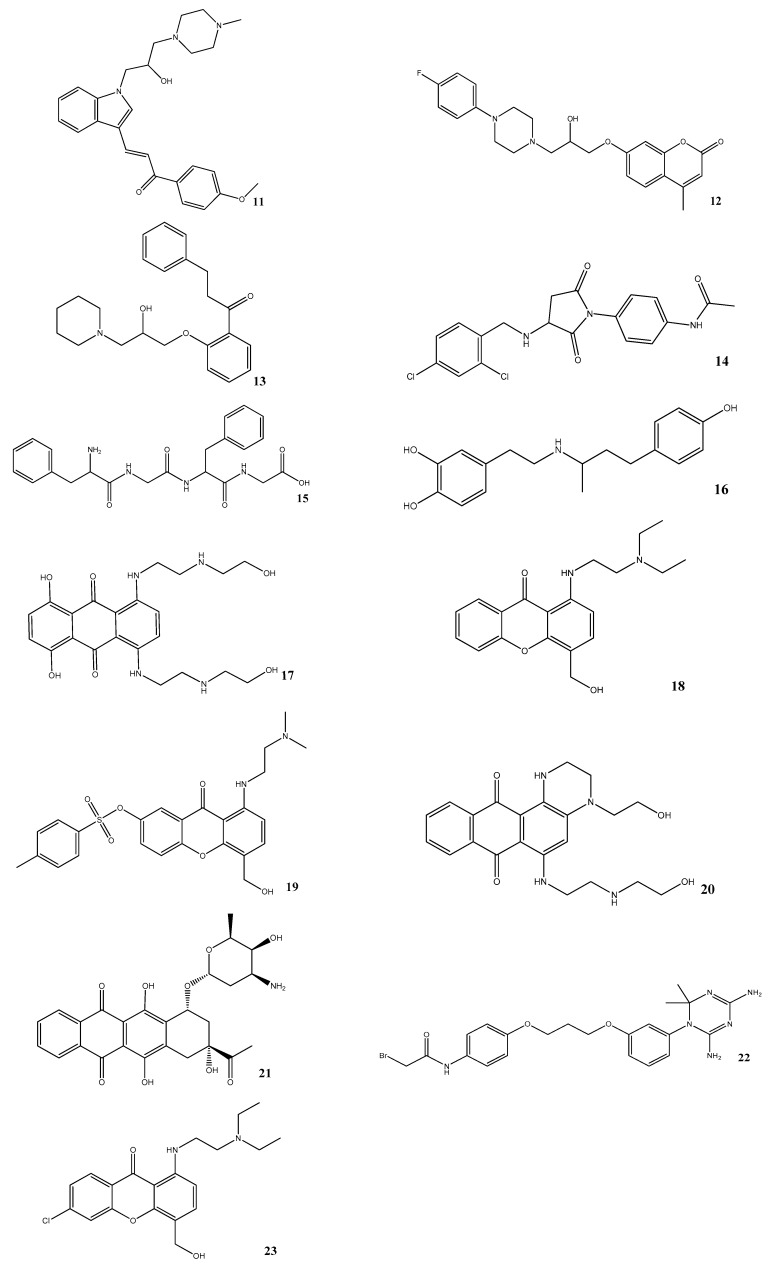
Chemical structures of the compounds identified herein as hit BACE1 inhibitors.

**Figure 8:**
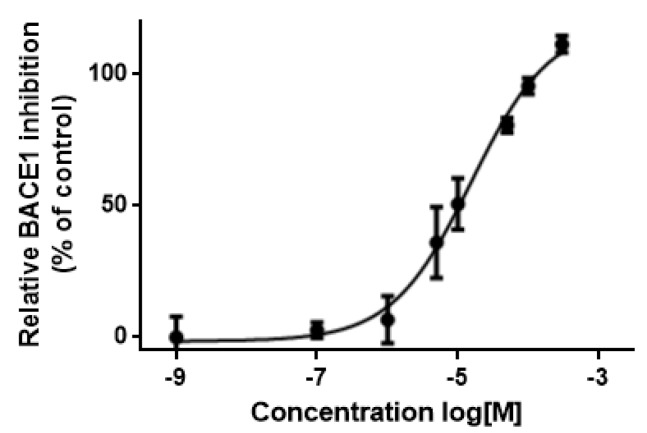
BACE1 inhibitory profile of the most promising hit-compound **11**. Each data point expresses the percentage of BACE1 activity inhibition and represents the mean ± SD of at least three independent experiments performed in triplicate (the control is the enzyme activity determined in the absence of inhibitors).

Lastly, thirty-four compounds were obtained for experimental evaluation. The results demonstrated that thirteen (compounds **11**–**23** in Figure 7) out of the thirty-four compounds displayed inhibitory effect against BACE1 (ranging from 16 to 50% of enzymatic inhibition). Their experimental data together with the associated physicochemical properties are shown in Table 4. Compound **11** presented the highest inhibitory activity on inhibiting BACE1 with an IC_50_ value of 15.4 ± 2.9 μM determined from the dose-response curve in Figure 8.

### 3.6. GOLD Binding Modes

**Figure 9:**
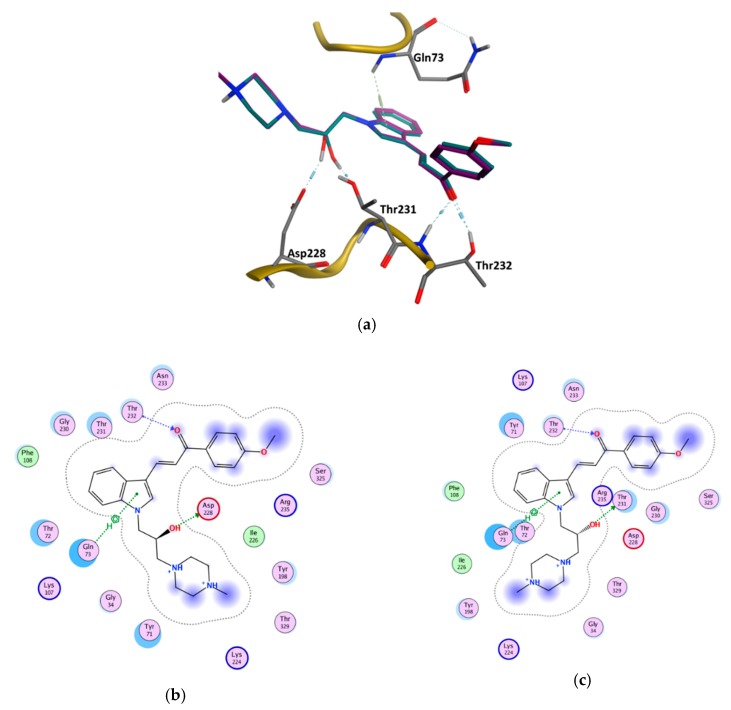
The binding modes of the two enantiomeric forms of compound **11** into the BACE1 active site (PDB ID: 2QP8), predicted by ChemPLP fitness function implemented in GOLD. (**a**) Superposition of the binding poses of (*S*)-enantiomer (purple) and (*R*)-enantiomer (blue). Highlighted amino acids are Gln73, Asp228, Thr231, and Thr232 involved in the ligand-protein interactions, the hydrogen bonding is depicted as blue dashed lines and aromatic interaction is colored in green. 2D ligand interactions diagram of (*S*)-enantiomer (**b**) and (*R*)-enantiomer (**c**). Green arrow represents sidechain acceptor; Blue arrow indicates backbone donor; Green dash lines with an arene symbol represents π-hydrogen interaction. The binding site residues are colored by their nature, with hydrophobic residues in green and polar residues in purple (blue and red contours indicate basic and acidic residues, respectively). The schematic pictures were generated using MOE.

Hit-compounds from Figure 7 are shown to interact with BACE1 active site mainly through hydrogen bonds and/or ionic attraction with Asp228, by hydrogen bonding with Thr72, Phe108, and Gly230, as well as with Thr231 and Thr232 through hydrogen bonds and/or aromatic interactions (Supplementary Figure S5). It is important to note that as the enantiomeric form of the indole-derivative **11** was not available neither by supplier’s information nor by Nuclear Magnetic Resonance (NMR) studies (although only a single enantiomeric form is present), docking studies were performed with the two enantiomeric forms—(*S*)- and (*R*)-enantiomer. The analysis of the predicted binding mode and the interactions within BACE1 binding site of the two enantiomeric forms is displayed in Figure 9 and Table 5. The hydroxyl group of the (*S*)-enantiomer and (*R*)-enantiomer forms a strong hydrogen bond with Asp228 (O-H·· O distance of 2.85 Å) and Thr231 (O-H·· O distance of 3.07 Å), respectively. These two residues act as sidechain proton acceptors. Moreover, the ketone group interacts with Thr232, which acts as a sidechain and backbone proton donor, by two strong hydrogen bonds ((*S*)-form: =O·· H-O distance of 2.91 Å and =O·· H-N distance of 2.90 Å; (*R*)-form: =O·· H-O distance of 2.94 Å and =O·· H-N distance of 3.03 Å). Additionally, the indole group interacts through a π-hydrogen interaction with the backbone amide of Gln73 ((*S*)-form: π·· H-N distance of 4.23 Å; (*R*)-form π·· H-N distance of 4.29 Å).

## 4. Discussion

In this study, a customized in silico-in vitro drug screening protocol was implemented to facilitate the identification of new compounds with BACE1 inhibitory activity. Firstly, two types of pharmacophore modelling approaches (SB and LB) were developed using MOE package. This method allows to characterize the structural features of molecules interacting with BACE1 and to define the spatial arrangement of pharmacophoric features [35]. The knowledge of BACE1-ligand 3D structures (there are more than 330 human BACE1 structures deposited in PDB [36]) and the ability of the ligands to favorably interact with BACE1 directed an approach based on the common ligand-receptor interactions. To generate an effective SB pharmacophore, the information about functional groups that interact with the target (noncovalent interactions’ type and interatomic distances) were considered, as well as the incorporation of information on the size and shape of the BACE1 binding site in the form of *ExcV*, the use of which is reported to provide more stringent and selective pharmacophore models [37]. The complexes used to generate the SB models were chosen based on the high resolution of the crystal structures and the structural variety of potent co-crystallized ligands, which represent different classes of BACE1 inhibitors [10]. Most notably, as the BACE1 enzyme is known to be quite flexible [38], this protocol considered multiple crystal structures for closed and semi-open conformations of BACE1 complexed with ligands displaying a wide range of size and shape that accounts for the inherent protein flexibility in the SB drug design approach (shown in Figure 3a). Besides, incorporating protein flexibility data is reported as improving the performance of the pharmacophore model [39]. Moreover, the PLIF application operates on the principle that the ligand-binding modes of the selected complexes are similar. Thus, the data in the homogeneous set of interaction fingerprints, which are considered important for activity, are conserved when converted in a homogeneous set of pharmacophoric feature points, further grouped and filtered according to which residues they interact with. The residues with a sufficiently tight grouping were converted into a pharmacophore query feature. Then, the query indicated by the application was refined by an iterative process of refinement and validation facilitated by the intimate connection between the Pharmacophore Search and the Pharmacophore Query Editor panels. The goal was to obtain a model with the best validation parameters, which resulted in the final three pharmacophores described in Table 1. These SB pharmacophore models share a *HBA* feature in the vicinity of Thr232, Asn233, Arg235, and Ser325, an aromatic center near Thr231 and Thr232, a *Hyd* feature into the hydrophobic cleft formed by Leu30, Phe108, Trp115, and Ile118 side chains and a *HBD&Cat* with a projected point representing the interaction between Asp228, among other features. We believe that these conserved interactions translated in pharmacophoric features are particularly important for receptor binding and ligands’ activity.

On the other hand, the method based on ligands exploits the knowledge of active compounds in order to retain information of common physical and chemical properties presumed to play a central role in target binding, which is essential for the bioactivity and the expected biological response [20]. The fundamentals of Elucidate application are based on the conception that all ligands bind to a receptor in a similar mode and there is enough diversity in the collection that the identified common functional groups, further converted in pharmacophoric features, are related to their activity [40]. Therefore, these features should highlight the important chemical features for BACE1 interaction. The training compounds for the model’s elucidation were chosen on the basis of a careful literature search, mainly focused on representing the broad range of scaffold classes of small molecules reported as potent BACE1 inhibitors [7] (Figure 2). These compounds represent the following classes: aminohydantoin (**1**), aminoimidazole (**2**, **10**), aminothiadiazine dioxide (**3**), aminooxazoline (**4**, **6**), aminothiazine (**5**, **8**), aminoisoindole (**7**), and aminopiperazinone (**9**). The selection of the pharmacophore models retrieved by the application focuses on the best fit values of the ligands to the respective query. This means that the LB models presented herein were generated through different alignment of the Training set structures. Similar to the SB approach, the refinement process followed an iterative cycle of refinement and validation in order to enhance the validation parameters. Lastly, the final three pharmacophores described in Table 2 were obtained. It is noted that all LB models share a *HBD* feature associated with one or two *PHBD*, reflecting the group presented in all compounds that acts as H-bond donor, which is expected to form H-bonding interactions with catalytic Asp32 and Asp228 (Supplementary Figure S4), crucial for their activity [9,10].

Accordingly, the most challenging aspect of this process is to obtain a highly selective pharmacophore model that detects the active compounds but discriminate the inactive compounds at the same time. Thus, in order to cautiously probe the quality of the proposed pharmacophore models, the following validation methods were used: (i) Training set to confirm that the ligands used to create the model are detected; (ii) Test set to discriminate the high active from moderate active compounds; (iii) Validation set with actives and decoys to evaluate the predictability of the hypothesis to pick the active from inactive compounds. Each pharmacophore modelling approach has a properly dataset used for model Testing validation purposes. It covers a broad range of structurally and chemically diverse molecules, representing the major part of the chemical space of known BACE1 inhibitors, with IC_50_ values from 0.3 nM to 1000 nM (Training and Test datasets are being distributed as part of the Supplementary Materials). The results present in Figure 4 showed general low hit detection in the Test set, especially for the SB models, which may reflect the diversity of the respective Training sets. As the LB approach presents more structural variety than the SB it is presumed to retrieve more Test set compounds. Furthermore, the performance of the pharmacophore modelling is more accurately estimated with larger data sets than smaller ones [27], thus, a validation set comprising a total of 18017 (276 active and 17741 decoys) molecules were used. Altogether, the best SB and LB model for the virtual screening were selected considering a careful balance on the sensitivity, specificity, accuracy, overall and early enrichment (Table 3) [28], as well as the results from the hit detection on Training and Test sets. Analyzing the validation data from the SB approach, no model stands out from the others. Although SB_Hyp2 has identified more hits in the Test set, SB_Hyp1 and SB_Hyp3 show more than 30-fold enrichment of top-ranked compounds, while SB_Hyp2 only shows 17-fold. As SB_Hyp1 model is more sensitive than SB_Hyp3 and presents the best TP vs. FP profile among all SB and LB models (Supplementary Table S1) it was indicated to VS. Regarding the LB approach, even though LB_Hyp1 does not present the best performance in the Test set detection, it is revealed to be the best model on the decoys validation, as well as in the enrichment of top-ranked compounds. It also displays the greatest TP vs. FP profile for the LB approach (Supplementary Table S1). Moreover, EF_0.5%_ is a very good indicator of the performance of the model, since it demonstrates the method’s ability to rank actives above decoys in the early portions of the hitlist. Succinctly, results showed that SB_Hyp1 and LB_Hyp1 successfully retrieved 37% and 38% of active compounds and are highly selective with values of 92% and 96%, respectively. Both models were found to perform with high accuracy (values of 91% and 95%, respectively), as well. In addition, SB_Hyp1 and LB_Hyp1 demonstrated a >4-fold and 8-fold overall enrichment over random for finding active molecules from chemical databases, respectively. Moreover, the analysis of ROC curves also assesses the performance of the hypotheses at discriminating active from inactive compounds. However, the hits that were not retrieved by the models are not scored by the application and were not considered. Still, as observed in the tables of Figure 5, the ROC plots show early recognition values for both methodologies. Indeed, SB_Hyp1 and LB_Hyp1 proved to be able to discriminate the active inhibitors from inactive or even low active compounds and could be effectively used to find novel anti-BACE1 compounds.

To this end, the pharmacophore models SB_Hyp1 and LB_Hyp1, depicted in Table 1 and Table 2, respectively, were employed to rapidly screen drug-like chemical libraries previously filtered by Lipinski’s Rule of Five [23]. As a criterion of the present study, compounds were required to satisfy both pharmacophores to significantly reduce the size of the database and to enhance the ability to detect promising hits. Around 716 hits were retrieved by the models, thereby yielding 0.12% of the initial screening dataset. In addition, GOLD docking was used to predict the binding conformation and classified the quality of the interactions of small molecules within the BACE1 binding site in order to form a stable complex [41]. These hits were further filtered using the predictive model BOILED-Egg for estimating the BBB penetration [33]. This will help decisions in the early stages of drug discovery for CNS targets. Thus, an effort was made that most compounds selected to be tested have BBB positive prediction (the only exception was for the high-scored NCI compounds). Therefore, driven by the top-scored results, thirty-four compounds were then evaluated using in vitro functional assays, whereby thirteen compounds (shown in Figure 7) exhibit a moderate inhibitory effect on BACE1. This is particularly true for compound **11** that displays an IC_50_ value of 15 μM, being the most promising BACE1 hit detected. In addition, the analysis of the BACE1 percent inhibition data and the respective GOLD scoring value of the thirty-four compounds tested (provided in Supplementary Table S2) reflects a feeble correlation between the docking scores of the top GOLD poses and experimentally measured activities. As was noted before, the intrinsic flexibility of the binding site of BACE1 may justify the low predictive power of the docking-based classification [9,42]. The highest docking score for these structures was 96.10, which is observed for compound **15** that displays an inhibitory activity of 30.7% at 10 μM, while the lowest docking score was 66.23, for hit **21**, which inhibits 18.1% of BACE1 activity at 10 μM (Supplementary Table S2 and Table 4). It is worth noting that there is a huge structural variability between the most promising compounds. For instance, compound **14** contains a pyrrolidine-2,5-dione (succinimide) moiety, compound **20** is a quinoxaline-derivative and compounds **18**, **19**, and **23** comprise the 9H-xanthen-9-one group. Importantly, to the best of our knowledge, none of these scaffolds have been reported before as BACE1 inhibitors. Interestingly, compounds **16**, **17**, and **21** are approved drugs for other pathological conditions, namely, dobutamine (β1 agonist catecholamine used in cardiogenic shock and severe heart failure), mitoxantrone (anthracenedione antineoplastic agent), and idarubicin (anthracycline antileukemic drug), respectively. Our approach also identified molecules with reported BACE1 inhibitors’ scaffolds, such as the peptidomimetic derivatives of the first-generation, of which **15** is an example, as well as other skeletons of small molecules from subsequent generations, such as indole-derivatives (**11**), coumarin-derivatives (**12**), piperazine-derivatives (**11**, **12**), piperidine-derivatives (**13**), and 1,3,5 triazine-derivatives (**22**) [10]. Noteworthy, these results revealed a strong validation of the protocol implemented herein. Moreover, as shown in Table 4, the majority of the hits (**11**–**14**, **16**, **18**, and **23**) are predictable to be central-acting compounds, which is a crucial property for being in vivo active against BACE1 and a possible drug for AD.

Additionally, as compounds **11** and **13** contain a chiral center, to determine which enantiomer was commercially acquired, these compounds were characterized by ^1^H-NMR, ^13^C-NMR, and 2D-NMR, which spectra showed that only an isomeric form was present for each sample (Supplementary Materials 2). Despite the NMR studies performed, it was not possible to identify the enantiomeric form evaluated in vitro. To predict which enantiomer of the most promising hit, compound **11**, is more stable at the binding site, the interaction profile of (*R*)- and (*S*)-**11** with the catalytic domain was obtained from docking studies. In fact, the analysis of the proposed interactions showed that both enantiomers establish important interactions with BACE1 binding pocket, displaying similar GOLD scores (91.70 for (*S*)- and 89.56 for (*R*)-enantiomer) (Figure 9 and Table 5). In both enantiomeric forms, residue Gln73 was involved in aromatic π-H interactions, Thr232 also being essential for the establishment of two strong hydrogen bonds. However, the hydroxyl group of the chiral center of (*S*)-enantiomer establishes a stronger hydrogen bond with Asp228 when compared to the interaction with Thr231 of the (*R*)-enantiomer. Concluding, the data showed that both enantiomers were able to interact with the catalytic domain via slightly different types of interactions, contributing to their binding stability. Additionally, the residues involved in these interactions are confirmed to play key roles in other reported small molecules with different scaffolds that bind to BACE1 [9].

## 5. Conclusions

In summary, this study reveals that the integration of computational methods with biochemical experiments is effective in the identification of novel hits acting as BACE1 inhibitors from relatively large databases. Regarding the molecular modelling approach, the protocol merges structure-based and ligand-based information through multiple steps for a fast and reliable virtual screening. The pharmacophore generation successfully followed two rational strategies: the first captures the protein-ligand essential interactions of BACE1-ligand complexes and the second was based on structurally diverse chemicals of known potent BACE1 inhibitors. These pharmacophore hypotheses were thoroughly validated and then used to rapidly screen large drug-like chemical libraries. Subsequently, docking simulations predicted compounds that may interact with relevant affinity within the BACE1 binding site. Afterwards, cell-free assays identified a collection of desirable hits (compounds **11**–**23** in Figure 7) that modulate BACE1 activity, with the most active compound showing low-micromolar binding affinity (compound **11** display an IC_50_ value of 15 μM). It is worth mentioning that these hit-compounds with new chemotypes may represent a starting point for further structural refinement in a hit-to-lead optimization process.

Indeed, in silico methods can assist AD drug discovery in rationally selecting compounds for in vitro screening that present a higher probability of being actives against known targets involved in pathological processes, namely BACE1, that is a relevant therapeutic target for AD.

## Figures and Tables

**Table 1:** Representation of SB pharmacophoric hypotheses and their inter-feature distances. Cyan sphere indicates the feature hydrogen bond acceptor (*HBA*); dark blue sphere represents hydrogen bond acceptor and metal ligation (*HBA&ML*); magenta sphere shows hydrogen bond donor and cation (*HBD&Cat*); red sphere represents hydrogen bond donor (*HBD*); dark magenta color represents projection of hydrogen bond donor (*PHBD*); orange sphere indicates aromatic (*Aro*); and green sphere represents hydrophobic (*Hyd*). The *ExcV* spheres were removed for better visualization.

Model	Features	Inter-Features Distance Range (Å)
**SB_Hyp1**	*HBA&ML; HBA; HBD&Cat; PHBD; Aro; Hyd;* 23*ExcV*	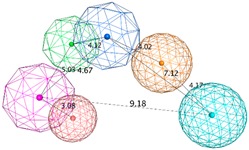
**SB_Hyp2**	*HBA; HBD&Cat; HBD; PHBD; Hyd; Aro*; 23*ExcV*	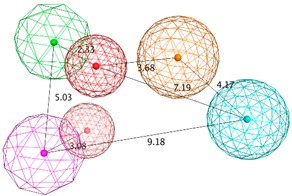
**SB_Hyp3**	*HBA&ML; HBA; HBD&Cat; HBD; PHBD; Hyd; Aro;* 23*ExcV*	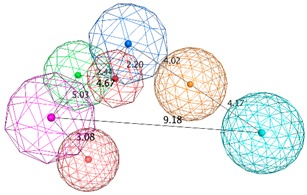

**Table 2:** Representation of LB (ligand-based) pharmacophoric hypotheses and their inter-feature distances. The alignment of multiple structurally different BACE1 inhibitors, the resulting pharmacophore features overlaid and the final derived pharmacophore are shown. The feature with the Cyan color indicates *HBA*; the dark blue sphere represents projection of hydrogen bond acceptor (*PHBA*); magenta color shows *HBD*; dark magenta color represents *PHBD*; orange color indicates *Aro*; green color represents *Hyd*; and dark green color denotes aromatic or hydrophobic center (*Aro/Hyd*). LB_Hyp1 and LB_Hyp3 were obtained from the Elucidate_1 strategy and LB_Hyp2 from the Elucidate_2 strategy.

Model	Original Features	Cover	Overlap Score	Refined Features	Inter-Features Distance Range (Å) and Molecular Alignment
**LB_Hyp1**	*Aro; PHBD; PHBA; HBD*	10	6.971	*HBA; PHBA; HBD;* 2*PHBD; Aro*	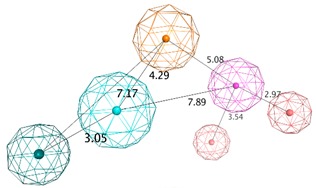
			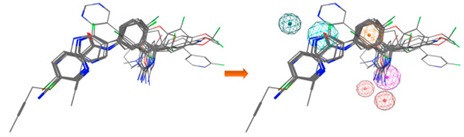		
**LB_Hyp2**	*Aro; PHBD; HBD; HBA*	10	6.826	*HBD;* 2*PHBD; Aro/Hyd; Aro; Hyd*	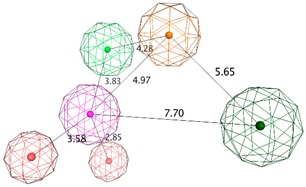
			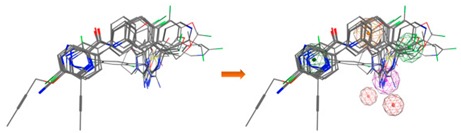		
**LB_Hyp3**	*Hyd; PHBD; PHBA; HBD; HBA*	10	6.899	*HBA; PHBA; HBD; PHBD; Aro; Hyd*	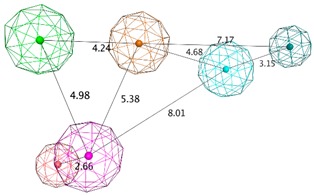
			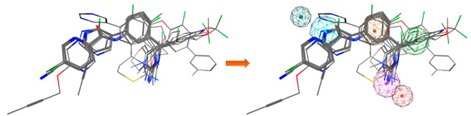		

**Table 3:** Pharmacophore model validation using decoys. D: total number of compounds in the dataset. A: total number of active compounds in the dataset. Ht: total number of compounds retrieved by the model. TP: true positive. TN: true negative. FN: false negative. FP: false positive. Se: sensitivity. Sp: specificity. EF: enrichment factor. EF_0.5%_: enrichment factor at 0.5% of the screened database.

Pharmacophore Models	D	A	Ht	TP	TN	FN	FP	% Se	% Sp	% Accuracy	EF_0.5%_	EF
**SB_Hyp1**	18017	276	1462	101	16380	175	1361	37	92	91	30.5	4.5
**SB_Hyp2**	18017	276	1206	94	16629	182	1112	34	94	93	16.7	5.1
**SB_Hyp3**	18017	276	595	78	17224	198	517	28	97	96	31.9	8.6
**LB_Hyp1**	18017	276	851	106	16996	170	745	38	96	95	22.5	8.1
**LB_Hyp2**	18017	276	1105	96	16732	180	1009	35	94	93	12.3	5.7
**LB_Hyp3**	18017	276	1774	93	16060	183	1681	34	91	90	13.1	3.4

**Table 4:** BACE1 percent inhibition data and physicochemical properties of the active hits. Average percent inhibition at 10 μM of the tested compounds. Data are present as the mean of at least three independent experiments performed in triplicate. Calculated in silico physicochemical properties of the compounds including molecular weight (MW), logarithm of the octanol/water partition coefficient (logP_o/w_) polar surface area (PSA), and the prediction of (blood–brain barrier) BBB penetration.

Compound	Compound Code	Average Percent Inhibition at 10 μM	MW	PSA	LogP_o/w_	BBB
11	AE-848/42798994	50.3	434.6	59.1	3.6	Yes
12	AP-124/43383636	33.5	413.5	63.4	2.8	Yes
13	AK-778/11348007	34.7	368.5	51.0	3.8	Yes
14	AN-919/15527216	31.3	407.3	83.1	2.9	Yes
15	NSC343027	30.7	426.5	155.1	0.4	No
16	NSC299583	29.9	302.4	77.3	3.7	Yes
17	NSC279836	22.6	446.5	172.3	−0.5	No
18	NSC166368	20.6	341.4	63.0	2.3	Yes
19	NSC354677	21.2	483.6	106.4	3.0	No
20	NSC270924	19.5	411.5	118.5	0.1	No
21	NSC256439	18.1	498.5	178.2	1.4	No
22	NSC109833	18.1	504.4	126.8	4.5	No
23	NSC166370	15.5	375.9	63.0	3.0	Yes
Reference compound	β-Secretase Inhibitor IV	52.9 *				

* average percent inhibition at 15 nM [34].

**Table 5:** Type of interactions of the two enantiomeric forms of compound **11** within BACE1 binding site. Residues Gln73, Asp228, Thr231, and Thr232 are involved in the ligand-protein interactions; Interactions’ energy (Kcal/mol) and docking score (ChemPLP score).

Enantiomer	Type of Interaction	ChemPLP Score
(*S*)	Gln73—Aromatic interaction −3.80 Asp228—Hydrogen bond interaction −3.30 Thr232—Hydrogen bond interaction −1.20 Thr232—Hydrogen bond interaction −1.80	91.70
(*R*)	Gln73—Aromatic interaction −3.40 Thr231—Hydrogen bond interaction −1.00 Thr232—Hydrogen bond interaction −1.00 Thr232—Hydrogen bond interaction −1.70	89.56

## Supplementary Materials

The following are available online at https://www.mdpi.com/2218-273X/10/4/535/s1, Figure S1: Training set of the SB pharmacophore modeling approach; Figure S2: Structure of the thirty-nine active set compounds against BACE1 used for the Test Set; Figure S3: 2D ligand-receptor interactions diagram of the PDB complexes used in SB pharmacophore approach; Figure S4: Overlap of the *HBD* and two *PHBD* features of the LB_Hyp1 model with the catalytic aspartic dyad of BACE1 and inter-features distance range (Å); Figure S5: Protein-Ligand Interaction Fingerprints (PLIF) analysis of the active compounds against BACE1; Table S1: Percentage of True Positive versus False Positive of pharmacophore models; Table S2: Scoring with GOLD and BACE1 percent inhibition data from the thirty-four in vitro tested compounds; Supplementary Material 2: ^1^H-NMR, ^13^C-NMR and 2D-NMR data for compounds **11** (AE-848/42798994) and **13** (AK-778/11348007).
