# Supplementary material for: Combining Virtual Screening Protocol and In Vitro Evaluation towards the Discovery of BACE1 Inhibitors
Source: Biomolecules. 2020 Apr 1;10(4):535. doi: 10.3390/biom10040535 (PMC7226079; doi:10.3390/biom10040535)
Supplement: Supplementary file 1 [file biomolecules-10-00535-s001.zip › supplementary 2 biomolecules-748549.docx]

Supplementary Material

Combining Virtual Screening Protocol and In Vitro Evaluation towards the Discovery of BACE1 Inhibitors

Judite R.M. Coimbra^1,2^, Salete J. Baptista^2,3^, Teresa C.P Dinis^2,4^, Maria M.C. Silva^1,2^, Paula I. Moreira^2,5^, Armanda E. Santos^2,4^, Jorge A.R. Salvador^1,2*^

1. University of Coimbra, Faculty of Pharmacy, Laboratory of Pharmaceutical Chemistry, 3000-548 Coimbra, Portugal; judite.coimbra@student.ff.uc.pt (J.R.M.C.); msilva@ff.uc.pt (M.M.C.S.); salvador@ci.uc.pt (J.A.R.S.)
2. CNC – Center for Neuroscience and Cell Biology, University of Coimbra, 3004-517 Coimbra, Portugal; saletejbaptista@gmail.com (S.J.B.)
3. Chem4Pharma, Edifício IPN Incubadora, 3030-199 Coimbra, Portugal;
4. University of Coimbra, Faculty of Pharmacy, Laboratory of Biochemistry, 3000-548 Coimbra, Portugal; tcpdinis@ci.uc.pt (T.C.P.D); aesantos@ci.uc.pt (A.E.S.)
5. University of Coimbra, Faculty of Medicine, Laboratory of Physiology, 3000-354 Coimbra, Portugal; pismoreira@gmail.com (P.I.M.)

- Correspondence: Jorge A.R. Salvador; salvador@ci.uc.pt; Tel.: +351 239 488 479;

**S2 FILE**

**^1^H-NMR, ^13^C-NMR and 2D-NMR data for compounds 11 (AE-848/42798994) and**

**13 (AK-778/11348007).**

NMR spectra were recorded using a Brucker Digital NMR-Avance 400 spectrometer and calibrated to residual solvent signals at δH 7.26 and δC 77.16 (CDCl3)

- 1. ^1^H-NMR spectrum for **11** recorded in CDCl₃

- 1. ^13^C-NMR spectrum for **11** recorded in CDCl₃

- 1. Dept135 spectrum for **11** recorded in CDCl₃

- 1. HMQC spectrum for **11** recorded in CDCl₃

- 1. HMBC spectrum for **11** recorded in CDCl₃

- 1. COSY spectrum for **11** recorded in CDCl₃

- 1. NOSY spectrum for **11** recorded in CDCl₃

- 1. ^1^H-NMR spectrum for **13** recorded in CDCl₃

- 1. ^13^C-NMR spectrum for **13** recorded in CDCl₃

- 1. Dept135 spectrum for **13** recorded in CDCl₃

- 1. HMQC spectrum for **13** recorded in CDCl₃

- 1. HMBC spectrum for **13** recorded in CDCl₃

- 1. COSY spectrum for **13** recorded in CDCl₃

- 1. NOSY spectrum for **13** recorded in CDCl₃
