# Supplementary material for: Combining Virtual Screening Protocol and In Vitro Evaluation towards the Discovery of BACE1 Inhibitors
Source: Biomolecules. 2020 Apr 1;10(4):535. doi: 10.3390/biom10040535 (PMC7226079; doi:10.3390/biom10040535)
Supplement: Supplementary file 1 [file biomolecules-10-00535-s001.zip › supplementary biomolecules-748549.docx]

Supplementary Materials

Combining Virtual Screening Protocol and In Vitro Evaluation towards the Discovery of BACE1 Inhibitors

Judite R.M. Coimbra^1,2^, Salete J. Baptista^2,3^, Teresa C.P Dinis^2,4^, Maria M.C. Silva^1,2^, Paula I. Moreira^2,5^, Armanda E. Santos^2,4^ and Jorge A.R. Salvador^1,2*^

1. University of Coimbra, Faculty of Pharmacy, Laboratory of Pharmaceutical Chemistry, 3000-548 Coimbra, Portugal; judite.coimbra@student.ff.uc.pt (J.R.M.C.); msilva@ff.uc.pt (M.M.C.S.)
2. University of Coimbra, Center for Innovative Biomedicine and Biotechnology (CIBB), Center for Neuroscience and Cell Biology (CNC), 3004-504 Coimbra, Portugal; saletejbaptista@gmail.com (S.J.B.)
3. Chem4Pharma, Edifício IPN Incubadora, 3030-199 Coimbra, Portugal;
4. University of Coimbra, Faculty of Pharmacy, Laboratory of Biochemistry and Biology, 3000-548 Coimbra, Portugal; tcpdinis@ci.uc.pt (T.C.P.D); aesantos@ci.uc.pt (A.E.S.)
5. University of Coimbra, Faculty of Medicine, Laboratory of Physiology, 3000-548 Coimbra, Portugal; pismoreira@gmail.com (P.I.M.)

* Correspondence: Jorge A.R. Salvador; salvador@ci.uc.pt; Tel.: +351 239 488 479;

Received: date; Accepted: date; Published: date

|   (**a**) |   (**b**) |
| --- | --- |
|   (**c**) |   (**d**) |

**Figure S1**. Training set of the SB pharmacophore modelling approach with an activity range (IC_50_ value) from 2.0 nM to 20.42 nM. (**a**) 2WF1–ChEMBL539436; (**b**) 2QMF–ChEMBL403727; (**c**) 2IRZ–ChEMBL219601; (**d**) 4ACU–ChEMBL3260842.

| ****  **ChEMBL3394224**  IC_50_ = 0.3 nM | | **ChEMBL3677276**  IC_50_ = 0.7 nM |
| --- | --- | --- |
| ****  **ChEMBL3667415**  IC_50_ = 0.73 nM |   **ChEMBL439521**  IC_50_ = 1.2 nM | |
| ****  **ChEMBL2152912**  IC_50_ = 2.0 nM | | ****  **ChEMBL3639946**  IC_50_ = 2.4 nM |
| ****  **ChEMBL3653708**  IC_50_ = 5.0 nM | | ****  **ChEMBL3673221**  IC_50_ = 6.0 nM |
|   **ChEMBL3672943**  IC_50_ = 6.6 nM | | ****  **ChEMBL3677269**  IC_50_ = 9.0 nM |
| ****  **ChEMBL1270334**  IC_50_ = 13.0 nM | |   **ChEMBL3978126**  IC_50_ = 15.0 nM |
| ****  **ChEMBL3640263**  IC_50_ = 16.0 nM | | ****  **ChEMBL3656139**  IC_50_ = 16.8 nM |
| ****  **ChEMBL533064**  IC_50_ = 20.0 nM | | ****  **ChEMBL1923753**  IC_50_ = 26.0 nM |
| ****  **ChEMBL2331708**  IC_50_ = 27.0 nM | | ****  **ChEMBL1957483**  IC_50_ = 31.6 nM |
| ****  **ChEMBL1923297**  IC_50_ = 40.0 nM | | ****  **ChEMBL3701711**  IC_50_ = 64.4 nM |
| ****  **ChEMBL598258**  IC_50_ = 80.0 nM | | ****  **ChEMBL2177905**  IC_50_ = 137.0 nM |
| ****  **ChEMBL237493**  IC_50_ = 140.0 nM | | ****  **ChEMBL3670919**  IC_50_ = 320.0 nM |
| ****  **ChEMBL3746896**  IC_50_ = 480.0 nM | |   **ChEMBL3688633**  IC_50_ = 499.5 nM |
| ****  **ChEMBL3656204**  IC_50_ = 521.1 nM | | ****  **ChEMBL3653345**  IC_50_ = 540.0 nM |
| ****  **ChEMBL1164612**  IC_50_ = 580.0 nM | | ****  **ChEMBL3650820**  IC_50_ = 661.0 nM |
| ****  **ChEMBL1271091**  IC_50_ = 690.0 nM | | ****  **ChEMBL253237**  IC_50_ = 700.0 nM |
| ****  **ChEMBL206442**  IC_50_ = 760.0 nM | | ****  **ChEMBL2403771**  IC_50_ = 760.0 nM |
| ****  **ChEMBL3703272**  IC_50_ = 827.0 nM | | ****  **ChEMBL607527**  IC_50_ = 950.0 nM |
| ****  **ChEMBL1821816**  IC_50_ = 970.0 nM | | ****  **ChEMBL1163225**  IC_50_ = 1000.0 nM |
| ****  **ChEMBL203120**  IC_50_ = 1,000.0 nM | |  |

**Figure** **S2**. Structure of the thirty-nine active set compounds against BACE1 used for the Test Set. These representative examples of diverse scaffolds reported as BACE1 inhibitors are shown with respective IC_50_ values.

| **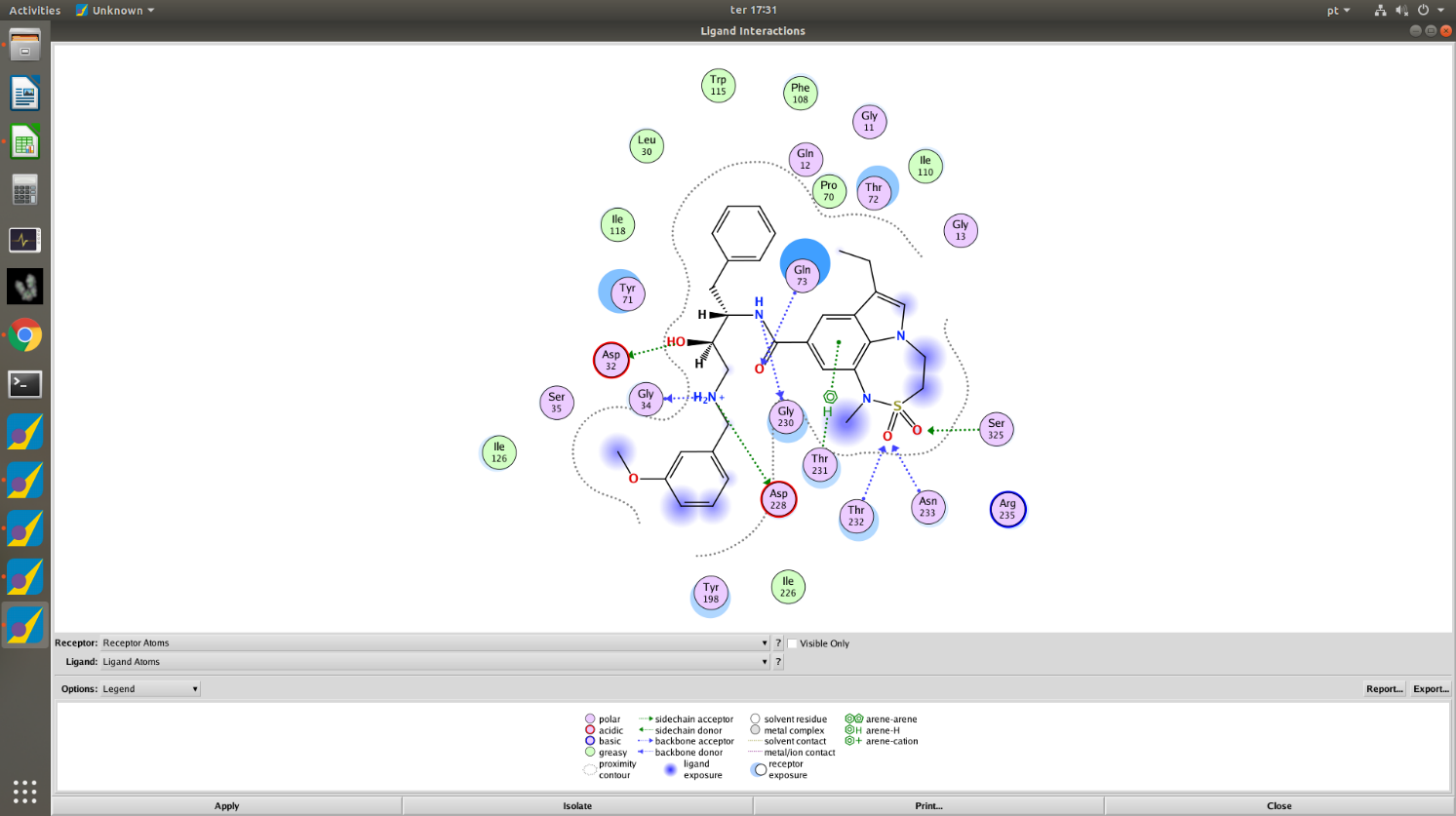**  (**a**) | **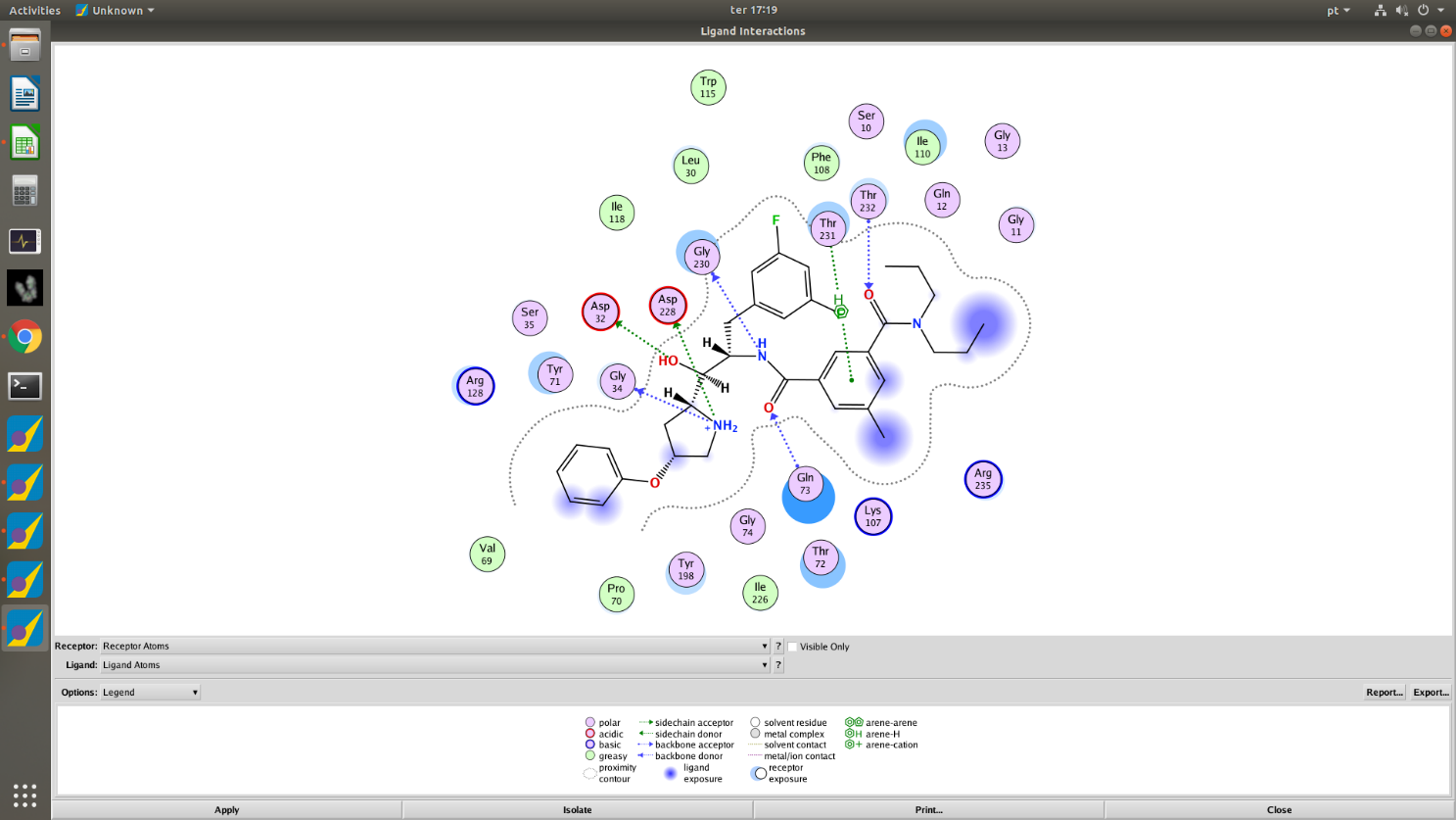**  (**b**) | |
| --- | --- | --- |
| 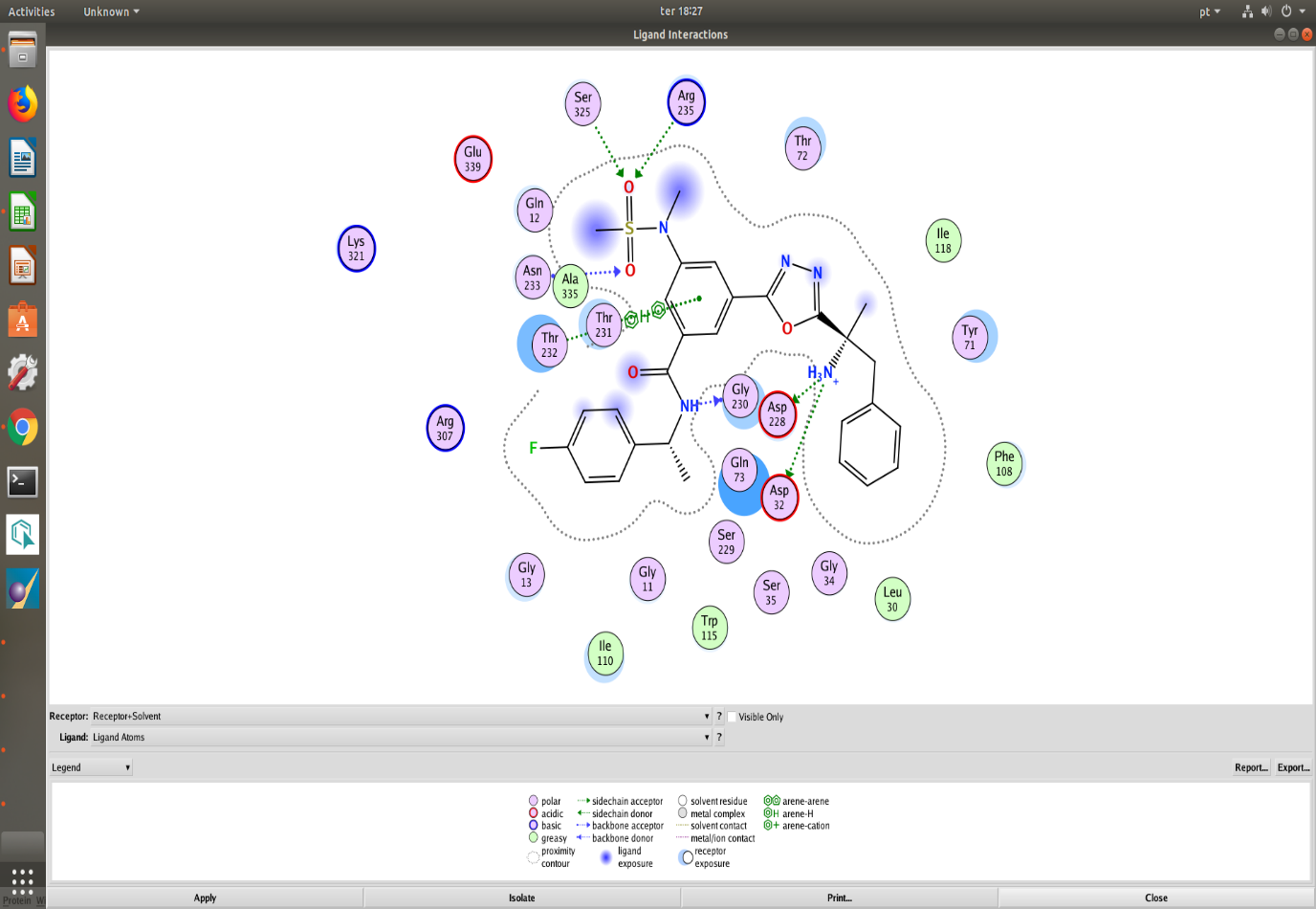  (**c**) | | 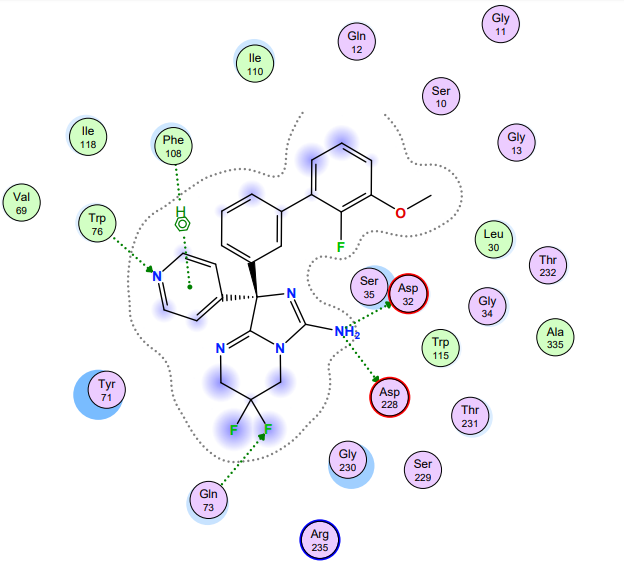  (**d**) |
| **Figure** **S3.** 2D ligand-receptor interactions diagram of the PDB complexes used in SB pharmacophore approach—ID: (**a**) 2WF1–CHEMBL539436; (**b**) 2QMF–CHEMBL403727; (**c**) 2IRZ–CHEMBL219601; (**d**) 4ACU–CHEMBL3260842. Green arrow represents sidechain acceptor or donor; Blue arrow indicates backbone acceptor or donor; Green dash line with an arene symbol represents π-hydrogen interaction. The binding site residues are coloured by their nature, with hydrophobic residues in green and polar residues in purple (blue and red contours indicate basic and acidic residues, respectively). The schematic pictures were generated using MOE. | | |

| **Table S1.** Percentage of True Positive versus False Positive of pharmacophore models. | | | | | | | | | |
| --- | --- | --- | --- | --- | --- | --- | --- | --- | --- |
| **SB_Hyp2** | | | | | | **LB_Hyp2** | | | |
| **% TP at 0.5% of FP** | **% TP at 1% of FP** | | **%TP at 5% of FP** | | **% TP at 6% of FP** | **% TP at 0.5% of FP** | **% TP at 1% of FP** | **%TP at 5% of FP** | **% TP at 6% of FP** |
| 9% | 14% | | 32% | | 34% | 7% | 11% | 29% | 35% |
|  |  | |  | |  |  |  |  |  |
| **SB_Hyp3** | | | | | | **LB_Hyp3** | | | |
| **% TP at 0.5% of FP** | | **% TP at 1% of FP** | | **%TP at 3% of FP** | | **% TP at 0.5% of FP** | **% TP at 1% of FP** | **%TP at 5% of FP** | **% TP at 12% of FP** |
| 17% | | 21% | | 28% | | 7% | 8% | 23% | 38% |

| 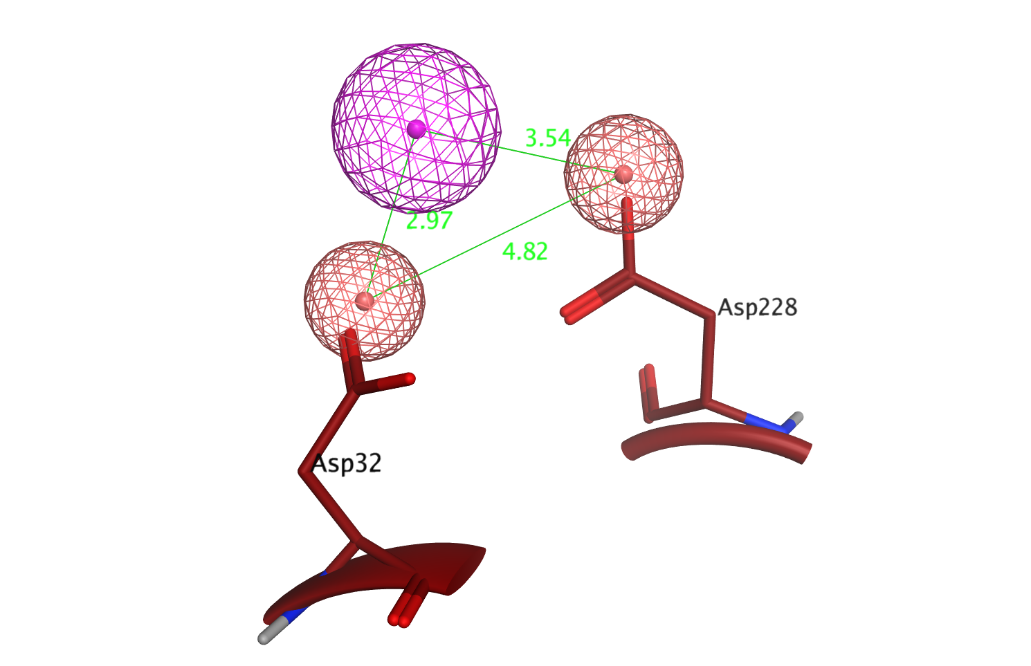 |
| --- |
| **Figure** **S4.** Overlap of the *HBD* (magenta colour) and two *PHBD* (dark magenta colour) features of the LB_Hyp1 model with the catalytic aspartic dyad of BACE1 and inter-features distance range (Å). |

| **Table S2.** Scoring with GOLD and BACE1 percent inhibition data from the thirty-four in vitro tested compounds. | | | |
| --- | --- | --- | --- |
| **In vitro tested compounds** | **Compound code** | **ChemPLP score** | **Average percent inhibition at 10 μM** |
|  | NSC343027 | 96.10 | 30.7 |
|  | NSC333463 | 93.03 | 13.4 |
|  | 7987192 | 92.35 | NE |
|  | NSC270924 | 91.49 | 19.5 |
|  | NSC279836 | 91.30 | 22.6 |
|  | AE-848/42798994 | 91.70/  89.56 | 50.3 |
|  | NSC354677 | 90.96 | 21.2 |
|  | NSC55229 | 90.89 | 12.1 |
|  | NSC278467 | 90.67 | 17.0 |
|  | LEG 17208331 | 87.15 | 14.8 |
|  | AN-465/43369843 | 86.46 | NR |
|  | NSC109833 | 86.16 | 18.1 |
|  | 7935938 | 85.48 | NE |
|  | SYN 22897555 | 85.05 | 7.2 |
|  | NSC299583 | 84.74 | 29.9 |
|  | NSC196473 | 84.30 | 12.4 |
|  | NSC81537 | 84.26 | 8.6 |
|  | NSC147850 | 83.80 | 8.7 |
|  | AP-124/43383636 | 83.00/  78.74 | 33.5 |
|  | SYN 15454460 | 82.71 | NE |
|  | NSC147843 | 82.34 | 9.6 |
|  | AK-778/11348007 | 82.02/  82.37 | 34.7 |
|  | NSC147841 | 81.96 | 13.0 |
|  | 7978733 | 81.78 | NE |
|  | 7661311 | 81.62 | NE |
|  | AG-205/33659009 | 81.54 | NR |
|  | NSC147840 | 81.00 | NR |
|  | 7364567 | 80.69 | NE |
|  | AN-919/15527216 | 75.71 | 31.3 |
|  | NSC147852 | 75.41 | 10.6 |
|  | ADD 17249371 | 73.61 | 7.7 |
|  | NSC166368 | 69.55 | 20.6 |
|  | NSC166370 | 68.40 | 15.5 |
|  | NSC256439 | 66.23 | 18.1 |
| Average percent inhibition at 10 μM of the tested compounds. Data are present as the mean of at least three independent experiments performed in triplicate.  NE—not effective.  NR—non-reproducible assay. | | | |

| **Population Histogram & Fingerprint Bit**  **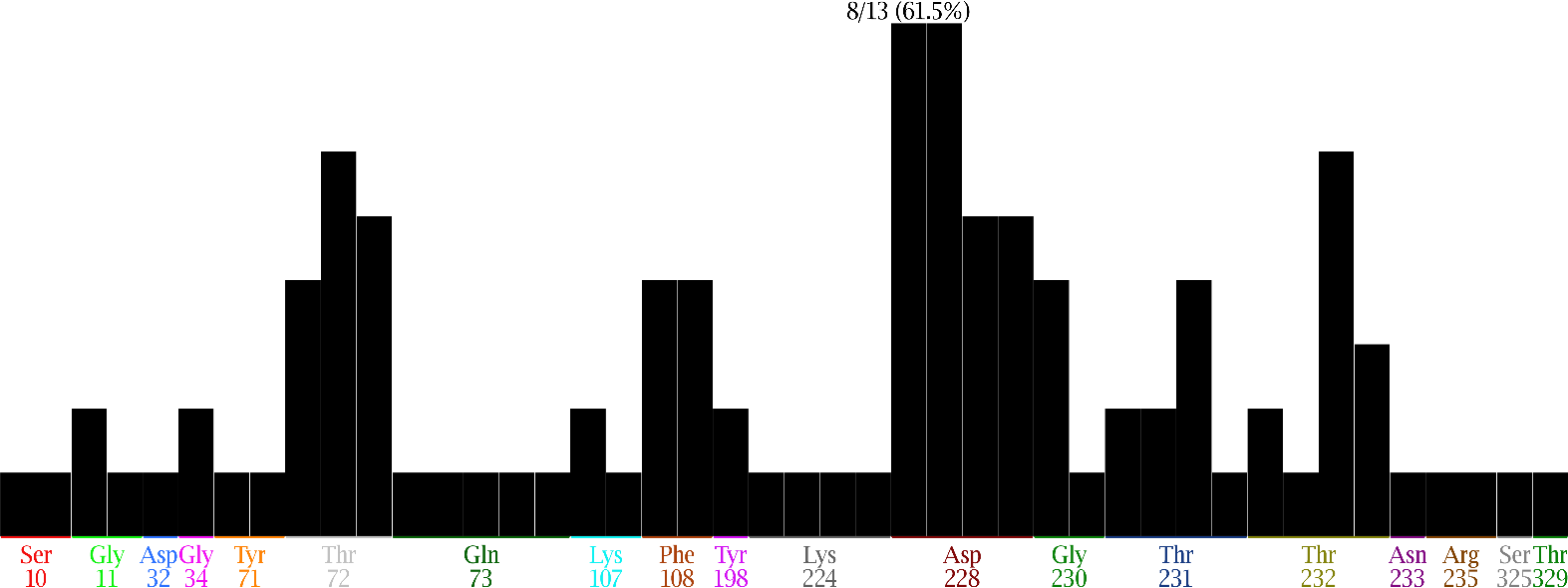**  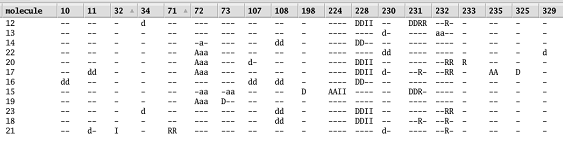  **(a)** |
| --- |
| **Ligand Display**  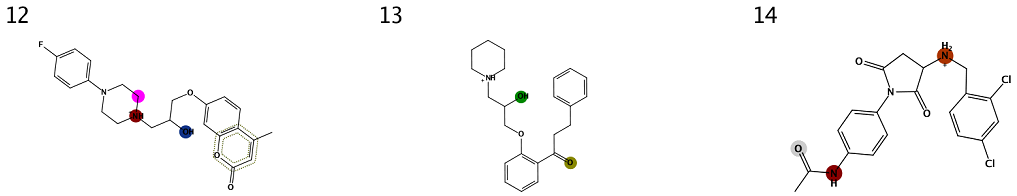  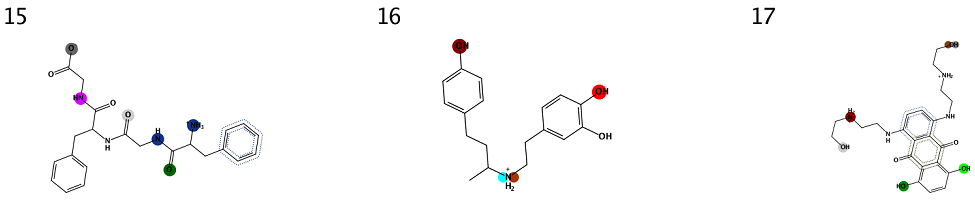 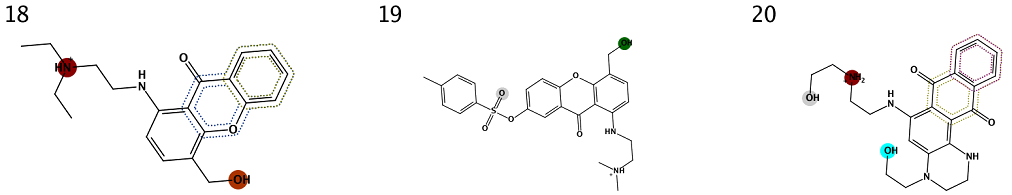  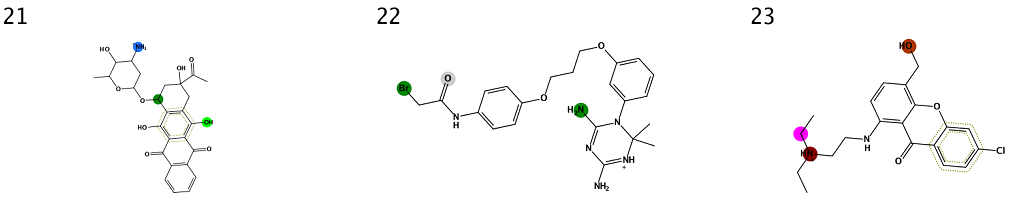  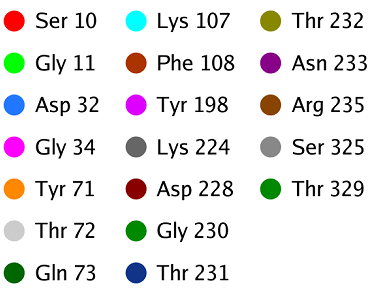  **(b)** |
| **Figure** **S5. Protein-Ligand Interaction Fingerprints (PLIF) analysis** **of the active compounds against BACE1.**  (**a**) **Population histogram of the 13 hit-compounds and Fingerprint bit.** The population histogram displays the frequency of occurrence of each fingerprint bit classified according to the residue of origin. The X-axis shows the residue numbers that correspond to each group of fingerprint bits, and is coded with an arbitrary sequence of colours. The Y-axis shows a black bar which shows the relative counts for the bits. At the top of the largest bar is shown the bit count, the number of entries involved (13), and the percentage of the entries which feature this bit (61.5%). Each character fingerprint bit means: - bit not set; D—sidechain hydrogen bond donor; A—sidechain hydrogen bond acceptor; d—backbone hydrogen bond donor; a—backbone hydrogen bond acceptor; I—ionic attraction; R—aromatic attraction.  (**b**) **Ligand Display.** The ligand graphic shows compounds **12-23**, in 2D diagrammatic form, with a concurrent alignment that is representative of bound conformation. Ligand atoms are annotated with a circle (those which correspond to a hydrogen bond), which is coloured in the same way as the X-axis for the population graphics. Surface contact interactions are annotated differently, as the number of ligand atoms affected is typically many; a dotted outline is drawn around the ligand atoms which are close to the residue. |
